# Mucopolysaccharidosis type VI phenotypes-genotypes and antibody response to galsulfase

**DOI:** 10.1186/1750-1172-8-51

**Published:** 2013-04-04

**Authors:** Marion M Brands, Marianne Hoogeveen-Westerveld, Marian A Kroos, Willemieke Nobel, George J Ruijter, Lale Özkan, Iris Plug, Daniel Grinberg, Lluïsa Vilageliu, Dicky J Halley, Ans T van der Ploeg, Arnold J Reuser

**Affiliations:** 1 Departments of Pediatrics and Clinical Genetics, Center for Lysosomal and Metabolic Diseases, Erasmus MC University Medical Center, Rotterdam, the Netherlands; 2 Departament de Genètica, Universitat de Barcelona, CIBERER, IBUB, Barcelona, Spain; 3 Department of Clinical Genetics, Center for Lysosomal and Metabolic Diseases, Erasmus MC University Medical Center, Dr Molewaterplein 50, 3015, GJ Rotterdam, The Netherlands

**Keywords:** Mucopolysaccharidosis, Maroteaux-Lamy syndrome, Lysosomal storage disorder, Genotype-phenotype correlation, Enzyme replacement therapy, Protein processing, Galsulfase

## Abstract

**Background:**

Mucopolysaccharidosis type VI (Maroteaux-Lamy syndrome; MPS VI) is an autosomal recessive lysosomal storage disorder in which deficiency of N-acetylgalactosamine 4-sulfatase (arylsulfatase B; ARSB) leads to the storage of glycosaminoglycans (GAGs) in connective tissue. The genotype-phenotype correlation has been addressed in several publications but the picture is not complete. Since 2007, enzyme-replacement therapy (ERT) has been available for patients with MPS VI in the Netherlands. The purpose of our study was to learn more about the genotype-phenotype correlations in MPS VI and the antibody response to ERT with galsulfase (recombinant human arylsulfatase B).

**Methods:**

We identified ARSB mutations in 12 patients and used site-directed mutagenesis to study their effect. Antibody levels to galsulfase were measured using ELISA and a semi-quantitative immunoprecipitation method. We assessed the *in vitro* inhibitory effect of antibodies on galsulfase uptake and their effect on clinical outcome.

**Results:**

Five patients had a rapidly progressive phenotype and seven a slowly progressive phenotype. In total 9 pathogenic mutations were identified including 4 novel mutations (N301K, V332G, A237D, and c.1142 + 2 T > C) together composing 8 pathogenic genotypes. Most mutations appeared not to affect the synthesis of ARSB (66 kD precursor), but to hamper its maturation (43 kD ARSB). Disease severity was correlated with urinary GAG excretion. All patients developed antibodies to galsulfase within 26 weeks of treatment. It was demonstrated that these antibodies can inhibit the uptake of galsulfase *in vitro*.

**Conclusions:**

The clinical phenotypes and the observed defects in the biosynthesis of ARSB show that some of the mutations that we identified are clearly more severe than others. Patients receiving galsulfase as enzyme-replacement therapy can develop antibodies towards the therapeutic protein. Though most titers are modest, they can exceed a level at which they potentially affect the clinical outcome of enzyme-replacement therapy.

## Background

Mucopolysaccharidosis type VI (Maroteaux-Lamy syndrome; MPS VI; MIM 253200) is a lysosomal storage disorder in which the deficiency of N-acetylgalactosamine 4-sulfatase leads to the storage of glycosaminoglycans (GAGs) in connective tissue. It is an autosomal recessive disease caused by mutations in the ARSB (arylsulfatase B) gene, which is located on chromosome 5q13-q14. More than 130 mutations have been identified, the vast majority being missense mutations [1]. Although the genotype-phenotype correlation has been described for several mutations, it is far from complete [2]. Patients with MPS VI present with connective-tissue pathology of varying severities; the main symptoms include restrictive pulmonary disease, cardiomyopathy, hepatosplenomegaly, corneal clouding, and bone disease with short stature. The variability in disease covers a clinical spectrum which is common in lysosomal storage disorder [1]. Since 2007, enzyme replacement therapy (ERT) has been available for patients with MPS VI in the Netherlands, where treatment and standardized care are concentrated at the national expertise centre at Erasmus MC University Medical Center. The effects of ERT on endurance, pulmonary function, hepatosplenomegaly, and GAG excretion were described in previous publications [3-5]. Antibody formation is common in patients receiving ERT for lysosomal storage disorders [6]. In Fabry disease and Pompe disease, for instance, it is a well-documented problem [7,8]. Due to the patient’s enzyme deficiency, the immune system apparently treats the recombinant human enzyme as a foreign protein. The extent of the antibody response may depend on whether ARSB is not produced at all, is misfolded and prematurely degraded, or finds its way to the lysosomes but lacks function, all of which depends on the mutations in the ARSB gene.

Here, we report on the genotypes and phenotypes of 12 Dutch patients with MPS VI, and on the correlation between genotypes, antibody response, and therapeutic effect of ERT.

## Methods

### Patients

Dutch patients with MPS VI are diagnosed, treated and followed-up by the Center for Lysosomal and Metabolic Diseases at Erasmus MC. Since 2007, twelve patients were referred to our center. Their diagnosis was based on a combination of clinical findings, arylsulfatase B deficiency in fibroblasts, and the presence of a pathogenic mutation in each of the two ARSB alleles. ERT treatment for MPS VI consists of 1 mg/kg recombinant human arylsulfatase B (galsulfase, Naglazyme®, BioMarin Corporation), administered by infusion once a week.

### ARSB activity

The ARSB activity in fibroblasts was spectrophotometrically measured on the basis of the conversion of p-nitrocatecholdisulfate to paranitrocatechol, as previously described, and is expressed in nmol paranitrocatechol liberated per mg cellular protein per hour [9].

### DNA analysis and site-directed mutagenesis

DNA was isolated from leukocytes or skin fibroblasts using routine procedures. After amplification, the exons and flanking intron regions of the ARSB gene were sequenced using an ABI 3730 DNA Analyser under standard GLP conditions. NM_32373.2 was used as reference sequence for the coding regions, whereby the ‘A’ nucleotide of the ATG translation-initiation codon at nucleotide position 560 constitutes +1 numbering of the cDNA sequence. The ATG-codon represents +1 for the amino acid numbering according to the galsulfase preprotein sequence NP_000037.2*.* The positions of intronic sequence variations were assigned on the basis of the cDNA sequence and the genomic contig sequence NC_000005.9, whereby the ‘A’ of the ATG start codon is at genomic position 78073032. The full length ARSB cDNA sequence cloned in expression vector pcDNA3 (p.V358; p.S384) was used as template for site-directed mutagenesis [10]. To introduce all mutations except splice-site mutations and established polymorphic sequence variations such as p.S384N into the wild-type ARSB cDNA, we used the QuickChange XL Site-Directed Mutagenesis Kit (Stratagene Life Technologies Co., La Jolla, CA, USA) for site-directed mutagenesis. In each case, the integrity of the resulting mutant constructs was confirmed by full-length sequencing of the ARSB cDNA insert.

Two independent colonies of each mutation were used to transfect COS-7 cells. The wild-type ARSB cDNA construct in pcDNA3 served as positive control, and a similar plasmid construct with the hexosaminidase-β cDNA as negative control. COS-7 cells were seeded into 6-well plates and grown overnight in Dulbecco’s modified Eagle medium (DMEM) (Lonza, Verviers, Belgium) supplemented with 10% fetal bovine serum (FBS), 50 U/ml penicillin, and 50 μg/ml streptomycin. Cells at more than 90% confluence were transfected with 2.0 μg plasmid using lipofectamine 2000 (InvitroGen). The cells were harvested 48 h and 72 hr later, and the ARSB activity was measured in the cell homogenates using paranitrocatecholdisulfate as substrate. The synthesis of ARSB was analysed by SDS-PAGE and immunoblotting using a polyclonal rabbit antiserum against recombinant human arylsulfatase B (galsulfase, Naglazyme®). In this assay, different molecular mass species reflect the synthesis of ARSB as 66 kD precursor, which is post-translationally modified to mature enzyme of 43 kD [11].

## Antibodies

### Immuno-precipitation

Blood samples for antibody titer determination were drawn just before the start of galsulfase infusions. Serum was prepared and stored at −80°C. Starting with a 25-fold dilution, two-fold serial dilutions were made in acetate buffer pH 6.0 (0.5 M sodium acetate, 10 mM barium acetate and 0.02% sodium azide) containing bovine serum albumin (BSA, Sigma) in a concentration of 0.2 mg/ml. A 10 μl solution of a 250-fold dilution of galsulfase (total activity approximately 0.3 μmol/hr) in acetate buffer was mixed with 10 μl diluted serum and 60 μl of a 1:6 suspension of Protein A Sepharose CL-4B beads in PBS. The mixture was incubated under continuous agitation for 1 h at room temperature. The beads were then removed by centrifugation (14,000 g), and the activity of galsulfase in the supernatant was measured with paranitrocatecholdisulfate.

### Pharmacokinetic (PK) analysis

Blood was drawn before the start of galsulfase infusion, and 1 h, 2 h and 2.5 h thereafter. It was collected again 15 min before the end of galsulfase infusion, at the end of the infusion, and 15, 30, 60 and 120 min thereafter. Plasma was prepared and stored at −80°C until use. To measure the percentage of antibody-bound galsulfase we used Sepharose beads, with and without Protein A bound to it, for precipitation as described in the preceding paragraph.

### ELISA

ELISA was used as a third method to determine antibody formation in response to ERT with galsulfase. A 96-well plate (Nunc, F96 Maxisorp, Denmark) was coated with 50 μL/well galsulfase in a concentration of 5 μg/mL diluted in phosphate-buffered saline (PBS, pH 7.4), and incubated for 2 hours at room temperature while shaking. Plates were blocked with 250 μL/well BSA/PBS (1 gram BSA/100 ml PBS from Sigma A7030), and incubated overnight at 4°C. After incubation, the plates were rinsed six times at room temperature with 200 μL washing buffer (0.5 mL Tween-20 per liter PBS). Plates were incubated with 50 μL of 5-fold serial dilutions of patients’ sera for one hour while shaking. Samples were diluted in dilution buffer (1 gram BSA and 50 μL Tween-20 per 100 mL PBS) in dilutions that ranged from 50-fold to 3,906,250-fold. A healthy person’s serum was used as a negative control, and rabbit antiserum prepared against galsulfase was used as a positive control. After washing, 50 μL conjugate was added to each well. For patients’ sera, we used polyclonal anti-human-[IgG, IgA and IgM]-HRP (Acris) in a 20,000-fold dilution. For polyclonal rabbit anti-serum, we used anti-rabbit-IgG-HRP (Sigma) in a 10,000 fold dilution. After washing, 100 μL Tetramethylbenzidine Microwell Peroxidase substrate (Kirkegaard and Perry Laboratories, Maryland) was added, and the plates were incubated for 10 minutes. The colorimetric reaction was stopped by adding 100 μL 1 M phosphoric acid (H_3_PO_4_). Absorbance was measured at 450 nm using a spectrophotometer (Thermo Electron corporation, Vantaa, Finland). The titer was determined as the maximal dilution at which absorbance was at least twice the absorbance of the negative control.

### Inhibition of galsulfase uptake

To assess the interference of antibodies with the uptake of galsulfase by human fibroblasts we added 160 μl plasma from patient 2, collected at peak-rate galsulfase infusion, to 640 μl culture medium (Ham’s F10 supplemented with 3 mM Pipes) in a 6-well-tissue-culture plate containing fibroblasts of this patient. In parallel, we added to other wells galsulfase in an amount equivalent to 2400 nmol/h ARSB activity in combination with either polyclonal rabbit anti-galsulfase antiserum or serum from a healthy individual. Uptake of galsulfase by the cells was measured 48 hours later.

### Clinical assessments

To evaluate the effect of ERT, we selected three variables: joint mobility (shoulder flexion), urinary GAGs and pulmonary function (Forced Vital Capacity, FVC). All assessments were performed according to standard protocol. The study protocol was approved by the Medical Ethical Committee at Erasmus MC University Medical Center.

## Results

### Patients

We describe 12 patients with mucopolysaccharidosis type VI in 10 unrelated families (Table 1). Five of the twelve patients had a rapidly progressive phenotype and presented before the age of 5 years with cardiomyopathy, dysmorphic facial features and/or severe joint limitations. The remaining seven patients had a slowly progressive phenotype. The diagnosis of all twelve patients was confirmed by the finding of ARSB deficiency in cultured skin fibroblasts, elevated GAG levels in their urines, and pathogenic mutations in both ARSB alleles. GAG values appeared to correlate with disease status. The pathogenic sequence variations comprised 7 missense mutations, 1 nonsense mutation and 1 mutation in the splice-donor site of exon 6. Four of these nine mutations were not previously reported. Eight of the 12 patients were the children of consanguineous marriages (third degree or higher) and were homozygous for the given mutations (Table 1).

**Table 1:** ARSB genotypes and phenotypes of 12 MPS VI patients

**Pt**	**Age at start of ERT**	**Age at diagnosis (years)**	**Main presenting symptom at diagnosis (years)**	**GAG at start of ERT (μg/mg creatinine)**	ARSB activity fibroblasts (nmol/h*mg)	**Allele 1 DNA^**	**Protein**	**Allele 2DNA^**	**Protein**	**Progression**	**Ethnicity**
1.	2.1	1.8	cardiomyopathy	941.6	85.7	c.903C > G c.1151G > A*	p.N301K p.S384N*	c.903C > G c.1151G > A*	p.N301K p.S384N*	Rapid	Turkish
2.	6.8	3.4	cardiomyopathy	1286.6	84.8	**c.1142 + 2T > C**		**c.1142 + 2T > C**		Rapid	Pakistani
3.	2.9	2.8	cardiomyopathy	554.4	32.3	**c.995T > G**	**p.V332G**	**c.995T > G**	**p.V332G**	Rapid	Marrocan
4.	2.3	1.9	macrocephalia	739.2	57.6	c.971G > T	p.G324V	c.971G > T	p.G324V	Rapid	Guinean
5.	8.3	7.8	joint abnormalities dysmorphic features	254.3	79.9	c.454C > T	p.R152W	c.454C > T	p.R152W	Slow	Turkish
6.^α^	18.3	10.1	joint abnormalities	105.6	46.7	c.454C > T	p.R152W	c.454C > T	p.R152W	Slow	Turkish
7. ^α^	7.6	0.7	positive sibling	230.6	61.9	c.454C > T	p.R152W	c.454C > T	p.R152W	Slow	Turkish
8.	10.6	10.2	joint abnormalities	192.7	50	c.629A > G	p.Y210C	c.937C > G	p.P313A	Slow	Dutch
9.	5.9	5.1	joint abnormalities dysmorphic features	206.8	38	c.629A > G	p.Y210C	c.979C > T	p.R327X	Slow	Dutch
10. ^β^	7.8	7.4	trigger fingers	213.8	40.7	c.629A > G	p.Y210C	c.979C > T	p.R327X	Slow	Dutch
11. ^β^	6.1	5.8	positive sibling	158.4	32.3	c.629A > G	p.Y210C	c.979C > T	p.R327X	Slow	Dutch
12.^#^	n.a.	2.8	joint abnormalities	712.8	57	**c.710C > A**	**p.A237D**	**c.710C > A**	**p.A237D**	Rapid	Iranian

Pt patient; α, β siblings; GAG glycosaminoglycans; ARSB Arylsulfatase B; ARSB activity, normal range 379–980 nmol/mg/hr; ^NCBI Reference sequence: NM_000046; * S384N has previously been described as a polymorphic change; novel mutations are marked in bold; #Pt 12 did not receive ERT in our hospital and is therefore only included in the genotype-phenotype section.

Except for patient 12, all patients are currently receiving ERT (galsulfase, Naglazyme®, 1 mg/kg weekly); three of them started before the age of 5 years. Only patient 2 has had infusion-associated reactions (IAR); these consisted of urticaria and general malaise.

### *Biosynthesis of* ARSB

ARSB is synthesized as a polypeptide of 533 amino acids that is co-translationally delivered into the lumen of the endoplasmic reticulum (ER) [12]. The 39 amino acids long N-terminal signal peptide is cleaved off, and the protein is glycosylated and folded. It attains a relative molecular mass of 66 kD, but is subsequently processed to a lysosomal form of approximately 43 kD with a central domain of 7 kD and a carboxyl-terminal domain of 8 kD [13].

To study the effect of the mutations in the ARSB gene, we attempted to visualize the synthesis and post-translation modification of ARSB in the patients’ fibroblasts by SDS-PAGE followed by Western blotting. However, due to the low intensity of the ARSB signal and the presence of ‘background’ staining, we failed to obtain clear results.

**Figure 1:**
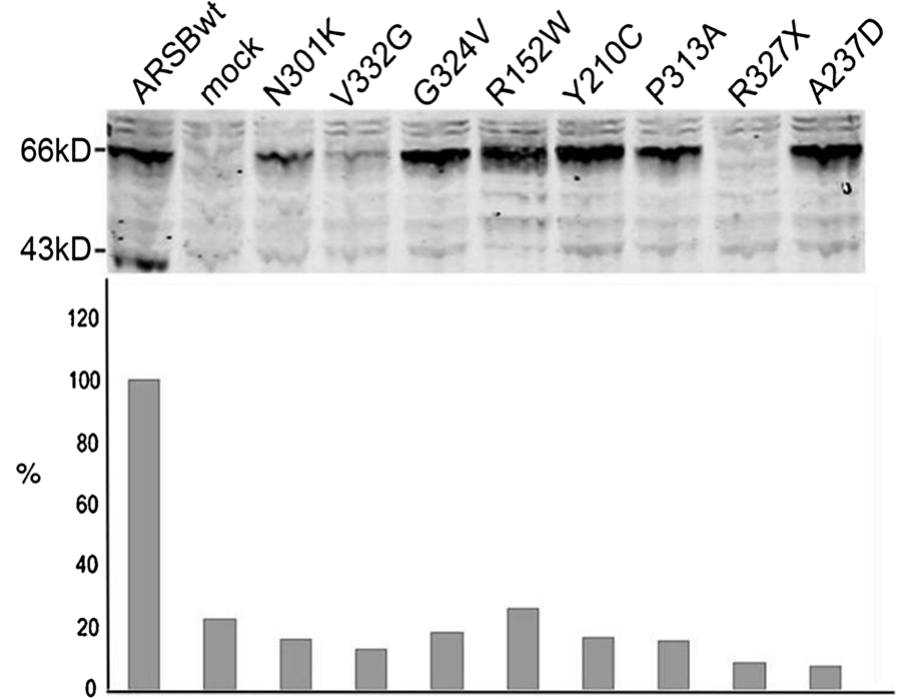
**Transient expression of wildtype and mutant ARSB cDNA constructs.** COS-7 cells were transfected with wildtype (wt) and mutant cDNA constructs. ‘mock’ indicates that the cells were transfected with a cDNA construct containing beta-hexosaminidase cDNA. SDS-PAGE followed by immunoblotting was performed and polyclonal rabbit anti-galsulfase serum was used to visualize ARSB. Equal amounts of protein were loaded per lane. The figure at the top shows the result of a representative experiment obtained at 72 hours after transfection. The figure at the bottom shows the ARSB activity as percentage of wild type ARSB activity as measured in cell homogenates at 48 hours after transfection.

As an alternative approach, we then used site-directed mutagenesis to introduce the mutations in the wild-type ARSB cDNA, and expressed these constructs transiently in COS-7 cells to gain an impression of how the mutations affected the biosynthesis of ARSB (Figure 1). None of the 7 missense mutations led to total loss of ARSB protein synthesis. The 66 kD precursor was detectable in all cases, but its amount varied from very little (V332G), to clearly less than normal (N301K), to near normal (G324V, R152W, Y210C, P313A, and A237D). By contrast, introduction of the nonsense mutation R327X led to total loss of ARSB production (Figure 1). Notably, expression of none of the missense mutations led to the formation of mature 43 kD ARSB protein, which was in our system best visualized at 72 hours after transfection (Figure 1 top, for ARSBwt), whereas the highest ARSB activity was measured at 48 hrs after transfection (Figure 1, bottom). At this time point the activity of R152W was barely above the ARSB activity in mock transfected cells, while all other mutants had less activity.

### Immune response to ERT

**Figure 2:**
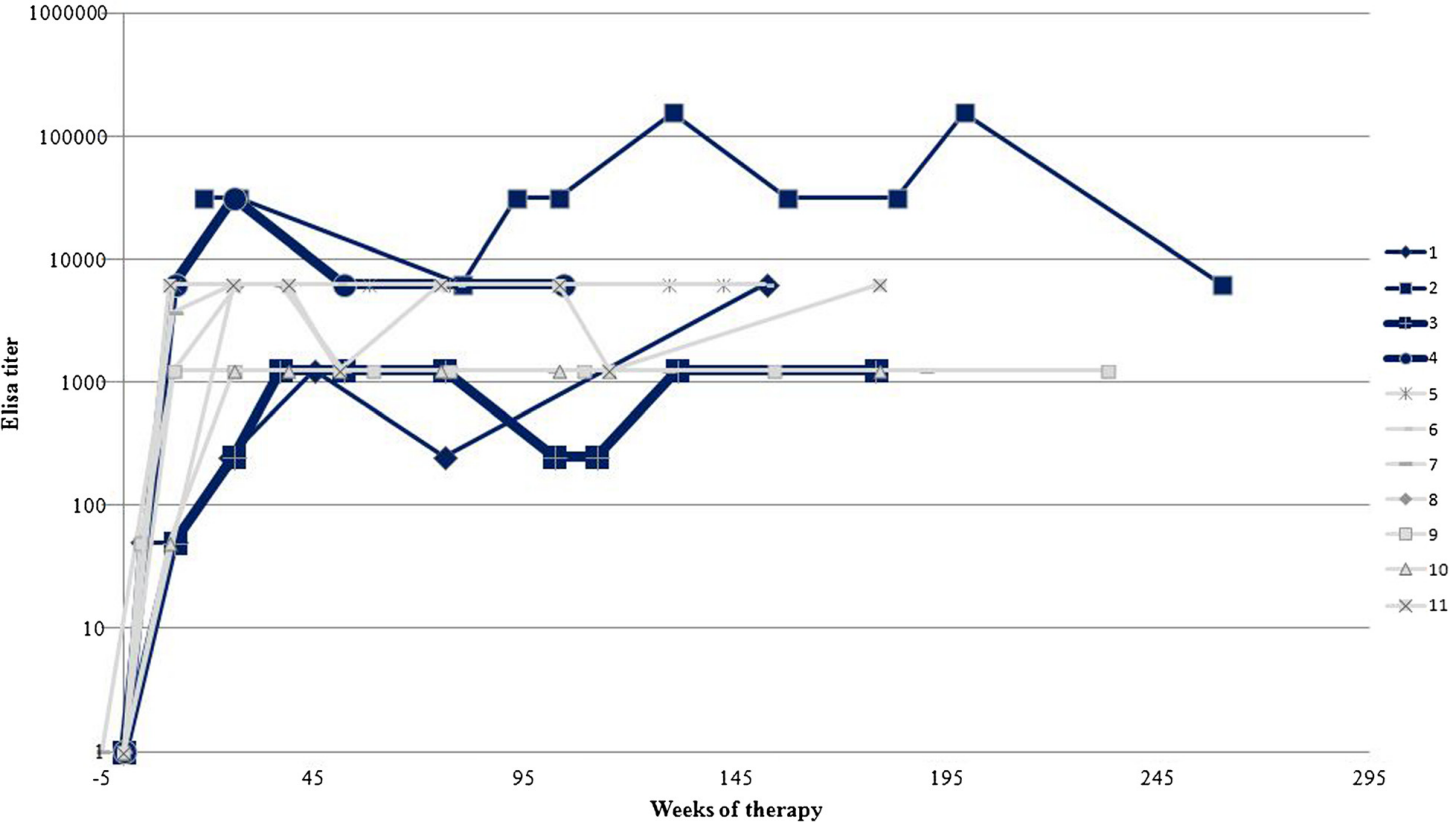
**ELISA titers of patients receiving ERT.** The black lines represent the patients with rapid disease progression; the grey lines represent the patients with a slower disease progression.

Within the first half year of ERT, all patients developed antibodies against galsulfase with an ELISA titer of at least 1:250 (range of 1:250 to 1:31,250) (Figure 2). After approximately three years of therapy, patient 2, who was homozygous for the splice-site mutation c.1142 + 2 T > C, had the highest antibody titer (1:156,250). Patient 4 had the second highest titer (1:31,250). Most patients had titers ranging from 1:1,250 to maximally 1:6,250, which remained fairly stable from one year after start of therapy until the end of follow-up.

**Figure 3:**
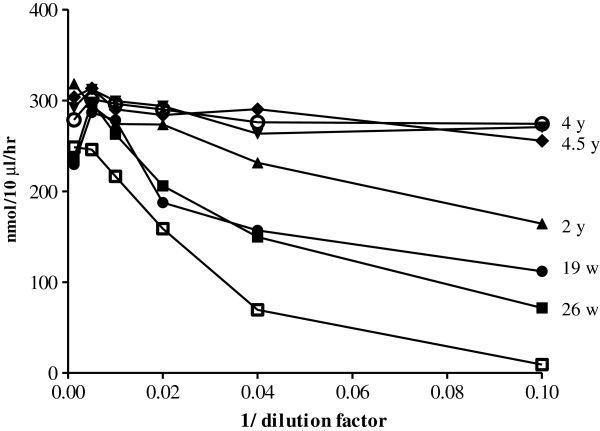
**Antibody titer of patient 2 by immunoprecipitation.** A fixed amount of galsulfase was incubated with serially diluted serum of patient 2. X-axis: 1/dilution factor (for example, 0.02 represents a dilution factor of 50). The antibody-bound galsulfase was precipitated using Protein A Sepharose beads, and the activity remaining in the supernatant was measured with paranitrocatecholdisulfate. The numbers at the right side of the curves represent the ERT duration in weeks (w) or years (y). The ELISA titers at these time points were 1:31,250 except at the 4y point (1:156,250). The open squares show the precipitation of galsulfase by rabbit anti-galsulfase serum; the open circles show the precipitation of a healthy individual (normal serum).

As an alternative method of estimating the height of the antibody titer, we performed an immunoprecipitation assay in which a given amount of galsulfase was precipitated from solution by serial dilutions of the patient’s serum. In this assay, only patient 2 had a measurable antibody titer (shown for patient 2 in Figure 3). This figure shows that the titer was highest after 26 weeks of therapy and then gradually declined over 4.5 years of follow-up. The result was somewhat unexpected, since the ELISA titer remained more or less constant over the same period (Figure 2).

**Figure 4:**
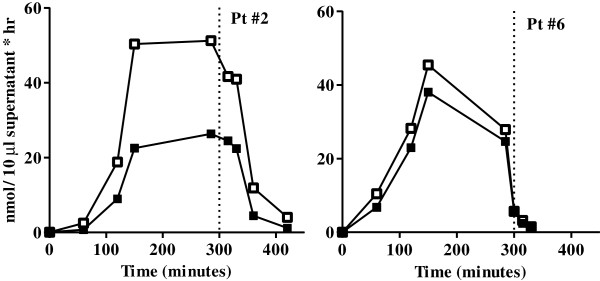
**Antibody binding of galsulfase during infusion.** Blood-plasma samples were collected from patient 2 and patient 6 just before start of galsulfase infusion (0 min) and at time points thereafter. The open symbols present the total galsulfase activity measured in the plasma; the closed symbols present the amount of activity remaining after Protein-A-Sepharose-mediated immunoprecipitation of antibody-bound ARSB. The difference between the curves represents the proportion of antibody-bound enzyme. The activity in the supernatant was measured with paranitrocatecholdisulfate and is expressed in nmol/10 μl per hour.

In addition to these two methods of determining the antibody titer, we also collected blood samples at regular intervals during the 5 hours of enzyme infusion in order to measure the capture of galsulfase by circulating antibodies. In patient 2 we performed this assay at 4.5 years after start of ERT, when her ELISA titer was 1:31,250; in patient 6, we performed it after 3.2 years of ERT, when his titer was 1:6,250. Figure 4 shows two curves for each of these patients. The curves with open symbols represent the total ARSB activity that was measured in the plasma, whereas the curves with closed symbols represent the galsulfase activity remaining after precipitation of antibody-bound galsulfase mediated by Protein A Sepharose. Thus, in patient 2, approximately 50% of galsulfase was antibody-bound after 2.5 hours (150 minutes) of infusion. The proportion of antibody-bound enzyme slightly decreased during infusion, and was 31% at the end of the infusion (300 minutes). In patient, 6 the amount of antibody-bound galsulfase was approximately 15% throughout infusion.

### Uptake of galsulfase by fibroblasts

To investigate the adverse effect that antibody-binding can have on the efficacy of ERT, we measured the uptake of galsulfase by cultured ARSB deficient fibroblasts. To this end we collected blood-plasma from patient 2 during infusion and selected the sample with the highest galsulfase activity. An aliquot of this sample was added to the cell culture medium. To other wells we added galsulfase together with either rabbit anti-galsulfase antiserum (rabbit serum) or serum from a healthy individual (normal serum).

After 48 hours, the galsulfase activity in the medium had dropped to 50% using normal serum and to 63% using the patient’s sample. Addition of rabbit serum resulted in a 50% drop of galsulfase activity within the first 15 minutes, but no further drop in activity was measured in the following 48 hours. At this time point, the cells were harvested and the intracellular galsulfase activity was measured. Uptake of galsulfase from the patient’s sample was 42% less than uptake of galsulfase combined with normal serum. The presence of rabbit antiserum inhibited the uptake of galsulfase almost completely (98%).

### Immune response and clinical outcome

**Figure 5:**
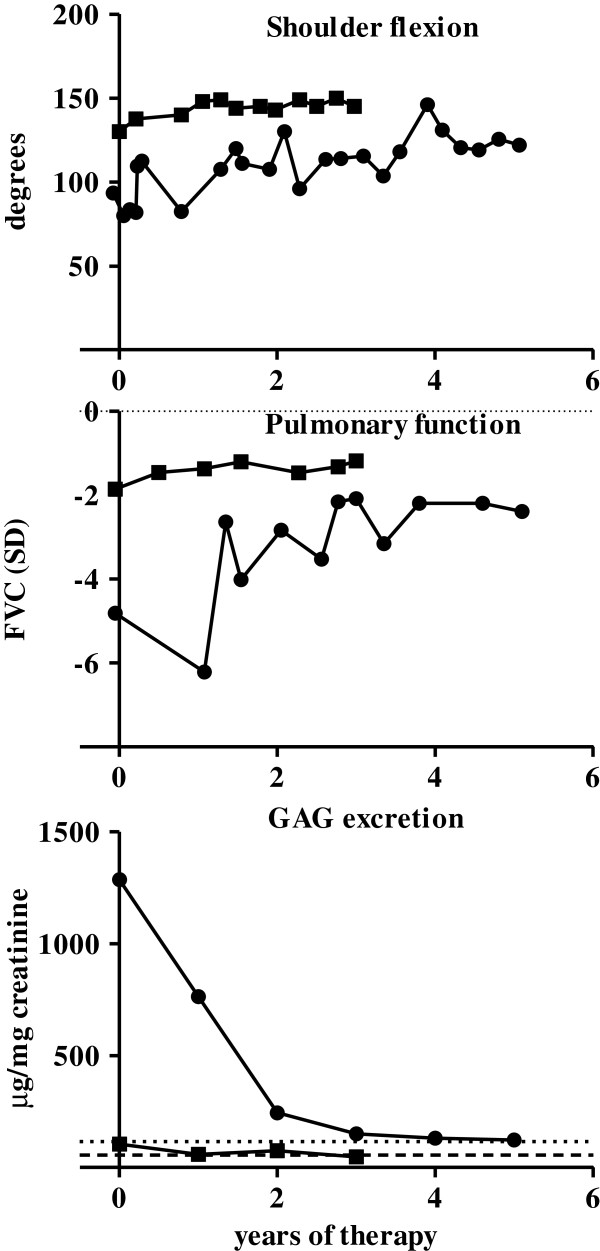
**Shoulder flexion, pulmonary function and glycosaminoglycan excretion.** During ERT, shoulder flexion (degrees), pulmonary function (FVC) and urinary glycosaminoglycan (GAG) excretion were measured in patient 2 (● ) and patient 6 (■). Shoulder flexion: normal range 175–180. Pulmonary function (FVC) is expressed as a Z-score of the predicted value. GAG excretion: the dashed lines denote the normal value for age (115 μg/mg creatinine for patient 2, and 55 μg/mg creatinine for patient 6).

Since the study group was very small and heterogeneous, we could not perform statistical analysis. Because analysis was further restricted by the limited availability and variability of clinical parameters, we could only investigate the overall correlation between immune response and clinical outcome. To this end, we compared the effect of ERT in two patients who had rather different immune responses: patient 2, whose antibody titers ranged from 1:31,250 to 1:156,250; and patient 6, whose titer was more or less constant at 1:6,250. Figure 5 shows the responses of both patients on FVC, shoulder flexion and GAG excretion. In both patients GAG values decreased significantly. GAG values remained slightly above the upper limit of normal in patient 2 over a period of 4.5 years. In patient 6, whose urinary GAG content had been lower at baseline than in patient 2, it normalized within 2.5 years of ERT.

Patient 2 had restrictive pulmonary disease at baseline (FVC −5.5 SD). During ERT, this improved from −4.8 SD −2.4 SD, though her respiratory capacity must also have been improved by the mitral valve replacement she underwent in the same period. In patient 6, FVC was −1.8 SD at baseline and −1.2 SD at last assessment. At start of therapy, both patients had severe joint restriction. In patient 2, the degree of shoulder flexion (starting from a 94 degrees restrictive shoulder flexion at baseline) improved by 30%; in patient 6, it improved by 12% (starting from 130 degrees restriction; normally 175–180 degrees). While the hepatomegaly and the splenomegaly both improved significantly under ERT in patient 2, no comparison was possible with patient 6, in whom these signs had not been evident at baseline. Neither could the effects of ERT on cardiac parameters be compared, as patient 2 received a mitral graft at the age of 7 years. Also the mild aortic regurgitation in patient 6 did not improve during ERT.

## Discussion

Since many pathogenic sequence variations have been identified in the ARSB gene, a genotype-phenotype correlation is slowly emerging [14-19] – a picture to which this description of our twelve patients and their genotypes now contributes. Five of our patients presented with rapidly progressive disease; progression in the other seven was slower. In total, we identified 9 different pathogenic mutations, 4 of which had not been published previously. Together they compose 8 different pathogenic genotypes. MPS VI is indeed very heterogeneous [13].

### Genotypes and phenotypes

Like others, we found that the level of residual ARSB activity in cultured fibroblasts using rather non-specific artificial substrates is a poor means of differentiating between patients with different clinical phenotypes. The radiolabeled three-saccharide substrates perform better [20,21]. By contrast, urinary GAG content seems to help indicating the degree of disease severity [12,22]. In our patient group too, a high urinary GAG content (mainly dermatan sulphate) of 300 μgram/mg creatinine or higher was associated with a severe phenotype.

With regard to our patients’ genotypes, 4 patients were homozygous for missense mutations and one was homozygous for a splice-site mutation. The advantage of homozygous genotypes over compound heterozygous genotypes is that the impact of the genotype does not need to be gathered from the additive effects of the two individual mutant alleles. Karageorgos et al. were able to correlate the genotype, urinary GAG excretion and phenotype of several patients with homozygous missense mutations [19].

Three of our patients were homozygous for p.R152W. The fact that they were the least severely affected patients in our study group is consistent with the literature indicating that patients with this mutation are not amongst the most severely affected. One publication about a 38-year-old woman describes this mutation as being associated primarily with a ‘cardiac phenotype’ [23]. While our three patients with this mutation do not have prominent cardiac signs, the oldest –who is currently 21 years old– does have mild aortic regurgitation. By contrast, four of the other missense mutations that we identified (p.N301K, p.V332G, p.G324V, and p.A237D) are severely pathogenic; this is suggested by the rapidly progressive phenotypes of the homozygotes in which they were found. Only p.G324V has been described previously, but in a compound heterozygote, meaning that its effect was still obscure [19]. Its great severity is indicated by the fact that our patient who is homozygous for it had macrocephalia, hepatosplenomegaly, severe joint deformities and eye problems at a very young age (2y old). As patient 2, who is homozygous for 1142 + 2 T > C, also had a rapidly progressive phenotype we conclude that this newly discovered mutation must be severe too. Moreover, this mutation affects the invariant splice donor sequence and is therefore expected to completely disrupt splicing [24]. Homozygosity for c.1142 + 1G > T in a patient with a very high GAG content in urine –like our patient– also resulted in total loss of ARSB protein [19]. Among the remaining patients, we identified four compound heterozygotes carrying the common p.Y210C mutation in combination with either p.P313A (patient 8) or p.R327X (patients 9, 10 and 11). p.Y210C is known to be associated with an intermediate MPS VI phenotype, which was also the case in our patients with this mutation [25]. Compound heterozygosity for p.Y210C and p.P313A was earlier described by Karakeorgos et al. in association with a slowly progressive phenotype [19].

The effect of 8 mutations was investigated by site directed mutagenesis and transient expression studies. Introduction of the non-sense mutation p.R327X in the wild type ARSB cDNA construct led to total absence of ARSB protein. The seven missense mutations had variable effects on the biosynthesis of ARSB; some resulted in (much) less than the normal amount of 66 kD precursor while others did not seem to have a dramatic effect on the amount of 66 kD ARSB as judged from several independent experiments. The low amount of 66 kD precursor in the respective cases might point to aberrantly folded proteins that are partly degraded by endoplasmic reticulum-associated degradation (ERAD) [12,26,27]. The expression of none of the 7 missense mutations in COS-7 cells led to the formation of both the 66 kD precursor as well as the 43 kD processed form of ARSB and none resulted in clearly measurable ARSB activity. This outcome agreed with the very low ARSB activity in fibroblasts of the patients. The lack of 43 kD ARSB by expression of p.Y210C was unexpected since Brooks et al. demonstrated in a pulse-chase experiment that 67% of p.Y210C-substituted ARSB is processed to mature 43 kD enzyme. However, the apparent discrepancy can relate to the fact that different methods and different antibodies were used (steady-state detection as opposed to pulse-chase labelling) [28].

### Antibody response to galsulfase

Within 26 weeks from the start of galsulfase infusions all patients showed seroconversion, which is consistent with observations during the clinical trials [4]. The ELISA titers differed between patients, varying during ERT from relatively low in some patients (1,250) to relatively high (1:156,250) in others.

Patient 2 consistently had the highest antibody titers and was the only patient who experienced infusion associated reactions (IARs). Notably, due to homozygosity for c.1142 + 2 T > C, the patient is not expected to have any level of endogenous ARSB expression, and is therefore immunologically naive to ARSB. This might underlie the strong immune response. Her high antibody titer as measured by ELISA was confirmed by immuno-precipitation. While the ELISA titer remained constantly high after 26 weeks of treatment, the titer obtained by immuno-precipiation declined. This would imply that the pool of antibodies directed against immobilized galsulfase (ELISA) remained constant while the pool of antibodies against native galsulfase decreased. The assay in which the pool of antibody-bound galsulfase is measured during enzyme infusion probably mimics the actual situation best; from it, we inferred that approximately 50% of galsulfase administered to patient 2 was captured by antibodies at the time that she had an ELISA titer of 1:31,250. In patient 6, whose ELISA titer was 1:6,250, the percentage of antibody-bound galsulfase was much lower.

With regard to the effect of antibody binding, we demonstrated for patient 2 that it can inhibit the uptake of galsulfase by cultured fibroblasts. It is plausible that a similar effect occurs *in vivo* and can limit the effect of ERT. In such a case, the adverse effect of antibody formation can possibly be overcome by higher dosing, unless higher dosing would lead to higher antibody titers or severe infusion-associated reactions.

We have no clinical indications that antibody formation in MPS VI has a very strong impact on the effect of ERT. Despite the much higher percentage of antibody-bound galsulfase during infusion in patient 2 compared to patient 6, both patients had similar increases in shoulder flexion, FVC and decrease in urinary GAG. It is known from other lysosomal storage diseases that immune response to ERT can reduce therapeutic outcome. In Pompe disease, for instance, antibody formation has been associated with loss of milestones and decreased pulmonary function [8]. In MPS VI, however, clinical changes in response to ERT are more subtle and the adverse effect of antibodies may go unnoticed [5]. We have investigated the effects of enzyme-replacement therapy in this patient group in a separate paper [29].

## Conclusions

In summary, our characterisation of the MPS VI patients currently receiving ERT in the Netherlands reflects considerable genetic heterogeneity. The clinical phenotypes and the defects we observed in the biosynthesis of ARSB show that some of the mutations we identified –known and novel alike– are clearly more severe than others. As in other lysosomal storage diseases, the antibody response to ERT can differ greatly between patients and may to some extent relate to the genotype [30]. Although antibody formation seems to have little impact on clinical outcome, we have demonstrated that the antibody concentration in the blood can reach a level at which it potentially affects the efficacy of ERT by inhibiting uptake of enzyme by the target tissues.

## Consent

Written informed consent was obtained from the patient for publication of this report and any accompanying images.

## Abbreviations

MPS VI: Mucopolysaccharidosis type VI; GAGs: Glycosaminoglycans; ARSB: Arylsulfatase B; ERT: Enzyme replacement therapy; pK analysis: Pharmacokinetic analysis; ELISA: Enzyme-linked immuno sorbent assay; SDS Page: Sodium dodecyl sulfate polyacrylamide gel electrophoresis; FVC: Forced vital capacity.

## Competing interests

We have no competing interests to declare. Financial support was obtained from the European Union, 7th Framework Programme ‘Euclyd – a European Consortium for Lysosomal Storage Diseases’ [health F2/2008 grant agreement 201678] and ‘ZonMw – Dutch organization for healthcare research and innovation of care [Grant 152001003 and 152001004]’.

## Authors’ contribution

MB carried out the immune assays and drafted the manuscript. MHW carried out the molecular genetic studies and helped drafting the manuscript. MK carried out the galsulfase uptake assays and critically revised the manuscript; WN participated in the molecular genetic studies and critically revised the manuscript; GR was responsible for the glycosaminoglycan studies and critically revised the manuscript; LO carried out the first ELISA experiments and critically revised the manuscript; IP participated in the design of the study and helped to draft the manuscript; DG provided the pcDNA3 construct and critically revised the manuscript; LV provided the pcDNA3 construct and critically revised the manuscript; DH participated in the mutation analysis and revised the manuscript; AvdP was involved in study design and assisted in writing the manuscript; AR participated in the study design, reviewed and revised all drafts. All authors have read and approved the final text and figures.
